# Classifying Cardiac Actin Mutations Associated With Hypertrophic Cardiomyopathy

**DOI:** 10.3389/fphys.2018.00405

**Published:** 2018-04-17

**Authors:** Evan A. Despond, John F. Dawson

**Affiliations:** Department of Molecular and Cellular Biology, Centre for Cardiovascular Investigations, University of Guelph, Guelph, ON, Canada

**Keywords:** actin, cardiovascular disease, hypertrophic cardiomyopathy, mutations, myosin, sarcomere, tropomyosin

## Abstract

Mutations in the cardiac actin gene (*ACTC1*) are associated with the development of hypertrophic cardiomyopathy (HCM). To date, 12 different *ACTC1* mutations have been discovered in patients with HCM. Given the high degree of sequence conservation of actin proteins and the range of protein–protein interactions actin participates in, mutations in cardiac actin leading to HCM are particularly interesting. Here, we suggest the classification of *ACTC1* mutations based on the location of the resulting amino acid change in actin into three main groups: (1) those affecting only the binding site of the myosin molecular motor, termed M-class mutations, (2) those affecting only the binding site of the tropomyosin (Tm) regulatory protein, designated T-class mutations, and (3) those affecting both the myosin- and Tm-binding sites, called MT-class mutations. To understand the precise pathogenesis of cardiac actin mutations and develop treatments specific to the molecular cause of disease, we need to integrate rapidly growing structural information with studies of regulated actomyosin systems.

## Introduction

Cardiovascular disease is the leading cause of death worldwide, and puts a strain on the global economy, specifically costing the Canadian economy >$22 billion annually (Gaziano, 2007; Smith, 2009). The end result of many cardiovascular diseases is heart failure and this can be influenced by a variety of factors, including genetics.

One such cardiovascular disease is hypertrophic cardiomyopathy (HCM), a group of related diseases characterized by hypertrophy of the ventricular myocardium, thought to be the result of increased calcium sensitivity. This disease can exhibit variable phenotypes, leading to difficulties in its clinical diagnosis (Baxi et al., 2016). Genes with mutations linked to HCM include myosin-binding protein-C (*MYBP3*), myosin heavy chain (*MYH7*), cardiac troponin T (*TNNT2*), and α-cardiac actin (*ACTC1*) (Seidman and Seidman, 2001; Walsh et al., 2017). Current research estimates that 1 in 200 individuals possesses an HCM-linked mutation (Semsarian et al., 2015).

A particularly interesting group of mutations is in the α-cardiac actin gene *ACTC1*. Due to the highly conserved nature of the actin sequence, the presence of mutations in *ACTC1* in patients with HCM is of note. To date, 12 *ACTC1* mutations have been identified in individuals with HCM, and 4 others in those with dilated CM (Olson, 1998; Mogensen et al., 1999, 2004; Olson et al., 2000; Van Driest et al., 2003; Morita et al., 2008; Olivotto et al., 2008; Kaski et al., 2009; Lakdawala et al., 2012).

The variety of genes linked to HCM encode proteins related to the sarcomere, the fundamental contractile unit of the heart, formed from interacting filaments of thin α-cardiac actin (ACTC) and thick β-myosin. These interactions are regulated principally by tropomyosin (Tm) and the troponin complex, which cooperate to reveal myosin-binding sites on the surface of actin filaments in the presence of calcium (Seidman and Seidman, 2001). The Mckillop and Geeves (1993) model of Tm action describes three states of Tm on F-actin in muscle (Lehman, 2017): (1) the blocked state, in which myosin-binding sites on actin are sterically blocked by Tm, (2) the closed state, where weak binding of myosin to actin is possible, and (3) the open state with strongly bound myosin present. Recent electron microscopy (EM) analyses largely agree with the states proposed by Mckillop and Geeves (1993) and reveal molecular interactions of the different states (Behrmann et al., 2012; von der Ecken et al., 2015, 2016; Gurel et al., 2017; Risi et al., 2017). With a growing understanding of the molecular interactions responsible for cardiac muscle contraction, rational explanations can be proposed for the impact of specific HCM-associated mutations in sarcomeric genes.

**FIGURE 1:**
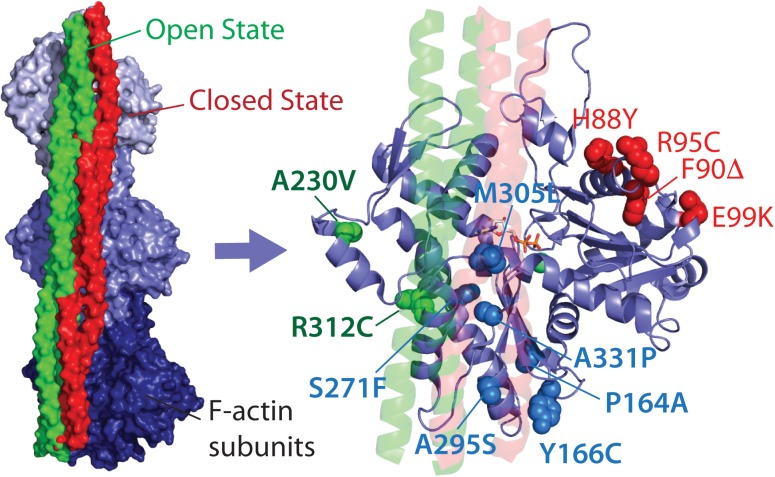
Location of HCM-linked actin mutations on the actin structure. Left, high-resolution structure of Tm bound to three subunits of one strand of F-actin in the open and closed states. Right, Location of Tm-class (T-class, labeled in green), myosin-class (M-class, labeled in red), and myosin and Tm-class (MT-class, labeled in blue) amino acid changes. Both images were produced using PDB 5N0J (Risi et al., 2017).

In this mini-review, we propose classification of the HCM-linked *ACTC1* mutations based on the location of the amino acid changes in ACTC, and hence their proposed protein interactions. We classify mutations that alter direct and exclusive interactions with the myosin motor protein as myosin- or M-class mutations, including the extensively studied E99K-ACTC variant, as well as H88Y, R95C, F90Δ, and S271F variants (**Figure 1**). Mutations that alter regulation of actin thin filaments by potentially disrupting interactions with Tm alone are termed Tm- or T-class mutations, including the A230V and R312C ACTC variants. Finally, mutations found in the Tm blocked state binding site that overlap with myosin binding are called MT-class mutations.

To date, the most thoroughly characterized mutation in any class is E99K-ACTC, while the remaining M-class mutations are largely uncharacterized (**Table 1**). Data exist for some T- and MT-class mutations, but further testing is required to provide a more comprehensive understanding of the molecular cause of HCM arising from *ACTC1* mutations.

**Table 1:** Summary of published research on M-, T-, and MT-class ACTC variants.

Class	Variant	Reference
Myosin/M	H88Y	**Morita et al., 2008**; Liu et al., 2017
	F90Δ	**Kaski et al., 2009**; Liu et al., 2017
	R95C	**Morita et al., 2008**; Liu et al., 2017
	E99K	**Olson et al., 2000**; Bookwalter and Trybus, 2006; Monserrat et al., 2007; Debold et al., 2010; Song et al., 2011, 2013; Mundia et al., 2012; Chow et al., 2014; Bai et al., 2015; Dahari and Dawson, 2015; Liu et al., 2017
	S271F	**Olivotto et al., 2008**
Tropomyosin/T	A230V	**Van Driest et al., 2003**; Mundia et al., 2012; Chow et al., 2014; Bai et al., 2015; Dahari and Dawson, 2015
	R312C	**Kaski et al., 2009**
Myosin and tropomyosin/MT	P164	**Olson et al., 2000**; Wong et al., 2001; Vang et al., 2005
	Y166C	**Mogensen et al., 2004**; Vang et al., 2005; Müller et al., 2012; Mundia et al., 2012; Chow et al., 2014; Dahari and Dawson, 2015
	A295S	**Mogensen et al., 1999**; Vang et al., 2005; Dahari and Dawson, 2015; Viswanathan et al., 2017
	M305L	**Mogensen et al., 2004**; Vang et al., 2005; Müller et al., 2012; Mundia et al., 2012; Chow et al., 2014; Dahari and Dawson, 2015
	A331P	**Olson et al., 2000**; Wong et al., 2001; Vang et al., 2005; Toko et al., 2010; Mundia et al., 2012; Bai et al., 2014; Chow et al., 2014; Dahari and Dawson, 2015

*Bold type denotes the original discovery of the mutation/variant.*

## M-Class Mutations

M-class mutations in ACTC are exclusive to sites of interactions with myosin, observed with loop 3 of the lower 50 kDa domain of myosin in actomyosin complexes as shown in recent EM work (Behrmann et al., 2012; von der Ecken et al., 2016; Banerjee et al., 2017; Fujii and Namba, 2017; Mentes et al., 2018). We also provisionally include the S271F-ACTC variant in the M-class based on its involvement in the high resolution structure of actomyosin structures (Gurel et al., 2017; Mentes et al., 2018).

### E99K

Originally described in a paper by Olson et al. (2000), the mutation encoding E99K-ACTC was found in several members of a family that exhibited HCM or similar cardiovascular conditions. The mutation was also found in 46 of 94 members of an unrelated family in Spain (Monserrat et al., 2007). E99K-ACTC displayed a significantly higher melting temperature and critical concentration (C_c_) compared to wild-type recombinant (WTrec) ACTC (Mundia et al., 2012). With unregulated filaments, the steady-state actin-activated myosin ATPase activity with E99K-ACTC protein showed a higher *K*_m_ compared to WTrec-ACTC, but no change in the *V*_max_ (Bookwalter and Trybus, 2006; Dahari and Dawson, 2015); there was also a decrease in the sliding velocity as measured by *in vitro* motility (IVM) assays (Bookwalter and Trybus, 2006; Debold et al., 2010; Dahari and Dawson, 2015; Liu et al., 2017). The binding affinity of E99K-ACTC for a short construct of myosin-binding protein-C (C0C2) was shown to be similar to WTrec-ACTC protein (Chow et al., 2014).

Further experiments examined E99K together with the regulatory proteins Tm and the troponin complex, forming regulated thin filaments (RTFs); these showed a significant decrease in maximum velocity, but no change in the concentration of calcium required to elicit a half-maximal response (pCa_50_ value) using IVM (Debold et al., 2010). E99K thin filaments isolated from patients or reconstituted *in vitro* showed greater calcium sensitivity with higher activation at lower levels of calcium (Bai et al., 2015), as well as impaired relaxation of fibers and a decrease in the number of motile filaments in IVM (Song et al., 2013). At the whole organism level, mice expressing E99K-ACTC had a higher mortality rate, ECG abnormalities, and generally mirrored the disease phenotype seen in humans (Song et al., 2011). Overall, the literature describing E99K-ACTC agrees with the hypothesis that an increase in calcium sensitivity and filament activation gives rise to the phenotype seen in both mice and humans.

### H88Y, F90Δ, and R95C

Three of the remaining M-class variants interact with loop 3 of myosin, and include H88Y, F90Δ, and R95C; these have only been examined as unregulated filaments to date. H88Y and R95C were both identified through genetic screening of pediatric patients with idiopathic cardiac hypertrophy (Morita et al., 2008). H88Y-ACTC produced subtle differences in myosin ATPase activity and sliding velocity *in vitro*, but no significant change from WTrec-ACTC; conversely, R95C-ACTC resulted in a significant decrease in myosin ATPase *V*_max_, but no difference in IVM measurements (Liu et al., 2017). F90Δ was first identified through genetic screening of 79 pediatric patients with HCM (Kaski et al., 2009), and had a significantly lower myosin ATPase *V*_max_ and faster IVM speeds compared to WTrec-ACTC (Liu et al., 2017). Further research with R95C, H88Y, and F90Δ RTFs is needed to determine if these variants have an impact on calcium sensitivity.

### S271F

The S271F-ACTC variant was discovered in a patient study examining myofilament-positive and -negative HCM (Olivotto et al., 2008). The S271 residue is distal to direct myosin and Tm-binding sites and is part of the hydrophobic plug of actin. To date, S271F-ACTC has not been characterized biochemically; however, recent high resolution structures of different myosin isoforms bound to F-actin (Gurel et al., 2017; Mentes et al., 2018) reveal the movement of the hydrophobic plug and interactions of the neighboring E270 that might be part of actomyosin function. For this reason, we provisionally assign the S271F-ACTC change to the M-class; whether S271F-ACTC modifies actomyosin interactions in cardiac muscle requires further experimentation.

## T-Class Mutations

Recent high resolution structures of F-actin with Tm show ACTC changes that are specific to the Tm-binding site, with others involved in both Tm and myosin binding (Behrmann et al., 2012; von der Ecken et al., 2015, 2016; Fujii and Namba, 2017; Risi et al., 2017; Mentes et al., 2018). T-class mutations include those changes on the ACTC protein that likely interfere exclusively with the binding of Tm.

### A230V

A230V was first identified in a study of 389 unrelated HCM patients (Van Driest et al., 2003). This change is removed from the myosin-binding site and is closer to the open state Tm site.

The A230V variant has a lower melting temperature, and a higher C_c_ (Mundia et al., 2012); there was no change in C0C2 binding compared to WTrec-ACTC (Chow et al., 2014). There were no significant changes in actomyosin interactions with the A230V-ACTC variant (Dahari and Dawson, 2015) in agreement with the distance of the change from the actomyosin-binding site. However, in reconstituted thin filaments, A230V-ACTC had decreased cross-bridge kinetics and pCa_50_ (Bai et al., 2015), showing increased calcium sensitivity and leading to hypercontractile sarcomeres. Together, these data suggest a generally less stable actin variant with impaired function in regulated systems.

### R312C

The R312C HCM-related variant is the most recent to be discovered, with an analysis of 79 unrelated pediatric patients revealing the mutation (Kaski et al., 2009). Direct interactions between Tm and R312 have been observed in high resolution structures (Risi et al., 2017). Changes at the R312 residue might alter local interactions seen with R288 of the *Dictyostelium* myosin-IE motor domain (Behrmann et al., 2012). Since no such interactions are observed in structures with myosin-II seen in striated muscle (Fujii and Namba, 2017), we include R312C as a T-class ACTC mutation.

To date, no primary research has been generated regarding R312C-ACTC. Interestingly, the R312H variant linked to dilated CM has been studied before (Olson, 1998; Wong et al., 2001; Debold et al., 2010; Mundia et al., 2012; Chow et al., 2014; Dahari and Dawson, 2015). The R312H actin variant displays consistent protein stability issues and, interestingly, results in a lower pCa_50_ of myosin activity with RTFs, with reduced maximum activation. The pCa_50_ curve for R312H actin in IVM assays (Debold et al., 2010) is similar to that of seen with the A230V variant in reconstituted sarcomeres (Bai et al., 2015). The resulting hypercontractility is thought to cause HCM, whereas the R312H variant is associated with DCM. Future work is needed to determine if the R312C variant exhibits similar effects on contraction.

## MT-Class Mutations

ACTC changes in the Tm-binding site, particularly the blocked state, and myosin-binding site make up the MT-class of ACTC mutations. All of the MT-class ACTC changes are located closer to Tm in the blocked state than in the open state. There may be synergistic negative impacts of MT-mutations as they affect both the regulation and development of force.

### Y166C

The Y166C-ACTC variant was discovered through mutational analysis of 206 unrelated HCM patients (Mogensen et al., 2004). The amino acid at position 166 is 14.4 Å from Tm in the blocked state and located in the hydrophobic pocket between subdomains 1 and 3 of actin. This region also binds sections of the lower 50 kDa domain of myosin through hydrophobic interactions (Fujii and Namba, 2017); however, Y166 participates primarily with the docking of the DNase-I-loop of the next actin subunit in F-actin to form stable filaments.

Y166C-ACTC has slightly more efficient folding than WTrec-ACTC (Vang et al., 2005), an increased polymerization rate, and a decreased G-actin ATPase rate (Müller et al., 2012). Müller et al. (2012) found that Y166C-ACTC had no difference in filament formation, a decrease in C_c_, and a decrease in actin-activated myosin ATPase rate and *V*_max_; our laboratory, however, showed a decrease in filament formation with Y166C-ACTC, an increase in C_c_, and no difference in myosin ATPase rate (Mundia et al., 2012; Dahari and Dawson, 2015). Since the same expression system was used for both sets of data with recombinant Y166C-ACTC protein, it is unclear why functional differences were observed. Y166C-ACTC was also shown to have a decreased affinity for C0C2 (Chow et al., 2014), but no altered interactions with other binding partners (Vang et al., 2005; Müller et al., 2012). While there appears to be some impact on F-actin stability, no major impact on actomyosin interactions has been reported with the Y166C variant (Müller et al., 2012; Dahari and Dawson, 2015) and research including regulatory proteins needs to be conducted.

### P164A

P164A was discovered alongside the E99K-ACTC variant and was found in a single patient identified with HCM (Olson et al., 2000). Given the proximity of P164 to Y166, the change from proline to alanine at this position might alter the local conformation of the actin protein, thereby influencing the interactions discussed for Y166. As a result, we place the P164A variant in the MT-class of mutations.

A study of the P164A variant in yeast actin (Wong et al., 2001) showed no significant change in intrinsic actin properties or interactions with myosin. Conversely, characterization of P164A-ACTC produced with *in vitro* translation suggested some structural instability with the protein (Vang et al., 2005). However, no characterization of P164A-ACTC with myosin alone or in regulated systems has been completed.

### A295S

A295S was the first ACTC variant to be found through clinical research and demonstrated that mutations in α-cardiac actin were linked to HCM. The mutation was discovered in 13 of 18 family members with familial HCM (Mogensen et al., 1999). The A295S position is among the closest to the Tm molecule in high-resolution structures (Behrmann et al., 2012; Risi et al., 2017), approximately 10 Å away. In addition, the A295 position on ACTC packs beside K328 of actin that forms electrostatic interactions with loop 4 of myosin in the actomyosin complex (Behrmann et al., 2012; Fujii and Namba, 2017).

The A295S variant exhibits no difference compared to WTrec-ACTC in several properties, including binding interactions, myosin ATPase and IVM activity, and filament incorporation (Vang et al., 2005; Dahari and Dawson, 2015). Viswanathan et al. (2017) created the only *in vivo* model of A295S-ACTC to date, using *Drosophila melanogaster* to express the variant in heart and flight muscle. Flies with cardiac expression of A295S-ACTC showed decreased relaxation, increased pCa_50_, and tension-generating periods, which led to hypercontractile muscle. When expressed in indirect flight muscle, flies had impaired flight due to physical tearing of the muscle as a result of destructive hypercontractility (Viswanathan et al., 2017). Therefore, the impact of the A295S change appears to be primarily at the level of contractile regulation. The association with loop 4 of myosin at this location is either not interrupted or of lesser significance to the overall binding of myosin.

### A331P

The A331P variant was discovered in one patient from the same study that identified E99K and P164A (Olson et al., 2000). A331 of ACTC interacts either directly with myosin (Behrmann et al., 2012) or through association with the neighboring P333, forming part of a hydrophobic interaction with the CM loop of myosin (Fujii and Namba, 2017). In addition, A331 lies within the blocked Tm-binding site (Risi et al., 2017), suggesting that part of the regulatory action of Tm is to inhibit the interaction of the CM loop of myosin with actin.

Some research suggests that the A331P change affects F-actin characteristics (Wong et al., 2001), while others find no significant impact (Vang et al., 2005; Mundia et al., 2012), perhaps as a result of different isoforms and expression systems. The A331P-ACTC variant has a decreased affinity for the C-terminal fragment of cardiac myosin-binding protein (C0C2) (Chow et al., 2014), but no change in interactions with other binding partners, including myosin S1 (Wong et al., 2001; Vang et al., 2005). There was also no difference in the actin-activated myosin ATPase activity (Dahari and Dawson, 2015), sliding speed, or the number of moving filaments compared to WTrec-ACTC by IVM (Wong et al., 2001; Dahari and Dawson, 2015). These data suggest that the A331P change does not significantly alter local interactions with the CM loop of myosin.

A331P RTFs show markedly reduced calcium sensitivity, contractility, and cross-bridge force, but no change in cross-bridge kinetics (Bai et al., 2014). Decreased calcium sensitivity indicates that the A331P change keeps Tm in the blocked state and is thought to be indicative of DCM; therefore, it is of interest that an HCM-associated change produces the opposite effect on calcium sensitivity.

The differences between WTrec- and A331P-ACTC in RTFs do not translate to an *in vivo* system, as a transgenic mouse expressing cardiac muscle A331P-ACTC failed to develop a HCM phenotype (Toko et al., 2010), but this may be due to the presence of an epitope tag or WT-ACTC protein during ectopic expression. There is a wealth of information regarding the A331P variant, but a lack of clear connection between the change and the development of HCM.

### M305L

M305L was discovered in one individual during the same clinical investigation that identified Y166C (Mogensen et al., 2004). Similar to A331 above, the M305 residue packs against P333 of ACTC; M305 is the closest of all HCM-associated ACTC variants to Tm, being less than 9 Å apart in the blocked state. Moreover, the M305 residue is part of the nucleotide-binding site of actin forming an essential structural component of the functional protein.

The association with the nucleotide-binding site of actin is reflected in changes in the intrinsic actin properties of the M305L variant, with increased Pi release and polymerization rates (Müller et al., 2012; Mundia et al., 2012). Conversely, it shows no difference in protein-binding interactions (Vang et al., 2005; Müller et al., 2012; Chow et al., 2014) or myosin ATPase activity (Dahari and Dawson, 2015). Given the proximity of M305 to Tm in the blocked state, it will be important to examine regulated systems to determine the mechanism of the M305L variant in HCM development.

## Conclusion

Hypertrophic CM is the most commonly inherited heart disease and contributes to the significant economic and healthcare burden of cardiovascular disease in society. Understanding the mechanistic cause of HCM has become increasingly important in research, with experiments moving beyond basic biochemical properties to investigate altered protein–protein interactions. Presented in this mini-review is a classification system for the identified α-cardiac actin mutations based on their proposed protein interactions (**Table 1**). Overall, all variants except S271F and R312C have been investigated at the biochemical level, but few have been thoroughly studied in higher-order systems. This situation leaves a gap in our current knowledge regarding the implications of ACTC variants in RTFs, and how changes in regulation translate to a disease state such as HCM. Closing this gap is critical for the development of new therapies that target specific protein interaction deficiencies, resulting in fewer side effects and greater quality of life for people living with heart disease.

## Author Contributions

JD took part in the research, wrote the first draft of the manuscript, edited and revised it, and formatted the final manuscript. ED revised the original manuscript, researching, restructuring, and reformatting the manuscript.

## Conflict of Interest Statement

The authors declare that the research was conducted in the absence of any commercial or financial relationships that could be construed as a potential conflict of interest.

## References

[B1] Bai F. Caster H. M. Dawson J. F. Kawai M. (2015). The immediate effect of HCM causing actin mutants E99K and A230V on actin-Tm-myosin interaction in thin-filament reconstituted myocardium. *J. Mol. Cell. Cardiol.* 79 123–132. 10.1016/j.yjmcc.2014.10.014 25451174

[B2] Bai F. Caster H. M. Rubenstein P. A. Dawson J. F. Kawai M. (2014). Using baculovirus/insect cell expressed recombinant actin to study the molecular pathogenesis of HCM caused by actin mutation A331P. *J. Mol. Cell. Cardiol.* 74 64–75. 10.1016/j.yjmcc.2014.04.014 24793351 PMC4264970

[B3] Banerjee C. Hu Z. Huang Z. Warrington J. A. Taylor D. W. Trybus K. M. (2017). The structure of the actin-smooth muscle myosin motor domain complex in the rigor state. *J. Struct. Biol.* 200 325–333. 10.1016/j.jsb.2017.10.003 29038012 PMC5748330

[B4] Baxi A. J. Restrepo C. S. Vargas D. Marmol-Velez A. Ocazionez D. Murillo H. (2016). Hypertrophic cardiomyopathy from A to Z: genetics, pathophysiology, imaging, and management. *Radiographics* 36 335–354. 10.1148/rg.2016150137 26963450

[B5] Behrmann E. Müller M. Penczek P. A. Mannherz H. G. Manstein D. J. Raunser S. (2012). Structure of the rigor actin-tropomyosin-myosin complex. *Cell* 150 327–338. 10.1016/j.cell.2012.05.037 22817895 PMC4163373

[B6] Bookwalter C. S. Trybus K. M. (2006). Functional consequences of a mutation in an expressed human α-cardiac actin at a site implicated in familial hypertrophic cardiomyopathy. *J. Biol. Chem.* 281 16777–16784. 10.1074/jbc.M512935200 16611632

[B7] Chow M. L. Shaffer J. F. Harris S. P. Dawson J. F. (2014). Altered interactions between cardiac myosin binding protein-C and α-cardiac actin variants associated with cardiomyopathies. *Arch. Biochem. Biophys.* 550–551, 28–32. 10.1016/j.abb.2014.04.003 24736382 PMC4306385

[B8] Dahari M. Dawson J. F. (2015). Do cardiac actin mutations lead to altered actomyosin interactions? *Biochem. Cell Biol.* 93 330–334. 10.1139/bcb-2014-0156 26194323

[B9] Debold E. P. Saber W. Cheema Y. Bookwalter C. S. Trybus K. M. Warshaw D. M. (2010). Human actin mutations associated with hypertrophic and dilated cardiomyopathies demonstrate distinct thin filament regulatory properties *in vitro*. *J. Mol. Cell. Cardiol.* 48 286–292. 10.1016/j.yjmcc.2009.09.014 19799913 PMC2813351

[B10] Fujii T. Namba K. (2017). Structure of actomyosin rigour complex at 5.2 Å resolution and insights into the ATPase cycle mechanism. *Nat. Commun.* 8:13969. 10.1038/ncomms13969 28067235 PMC5227740

[B11] Gaziano T. A. (2007). Reducing the growing burden of cardiovascular disease in the developing world. *Health Aff.* 26 13–24. 10.1377/hlthaff.26.1.13 17211010 PMC2365905

[B12] Gurel P. S. Kim L. Y. Ruijgrok P. V. Omabegho T. Bryant Z. Alushin G. M. (2017). Cryo-EM structures reveal specialization at the myosin VI-actin interface and a mechanism of force sensitivity. *Elife* 6:e31125. 10.7554/eLife.31125 29199952 PMC5762158

[B13] Kaski J. P. Syrris P. Esteban M. T. Jenkins S. Pantazis A. Deanfield J. E. (2009). Prevalence of sarcomere protein gene mutations in preadolescent children with hypertrophic cardiomyopathy. *Circ. Cardiovasc. Genet.* 2 436–441. 10.1161/CIRCGENETICS.108.821314 20031618

[B14] Lakdawala N. K. Funke B. H. Baxter S. Cirino A. L. Roberts A. E. Judge D. P. (2012). Genetic testing for dilated cardiomyopathy in clinical practice. *J. Card. Fail.* 18 296–303. 10.1016/j.cardfail.2012.01.013 22464770 PMC3666099

[B15] Lehman W. (2017). Switching muscles on and off in steps: the mckillop-geeves three-state model of muscle regulation. *Biophys. J.* 112 2459–2466. 10.1016/j.bpj.2017.04.053 28552313 PMC5479050

[B16] Liu H. Henein M. Anillo M. Dawson J. F. (2017). Cardiac actin changes in the actomyosin interface have different effects on myosin duty ratio. *Biochem. Cell Biol.* 96 26–31. 10.1139/bcb-2017-0136 28972856

[B17] Mckillop D. F. Geeves M. A. (1993). Regulation of the interaction between actin and myosin subfragment 1: evidence for three states of the thin filament. *Biophys. J.* 65 693–701. 10.1016/S0006-3495(93)81110-X 8218897 PMC1225772

[B18] Mentes A. Huehn A. Liu X. Zwolak A. Dominguez R. Shuman H. (2018). High-resolution cryo-EM structures of actin-bound myosin states reveal the mechanism of myosin force sensing. *Proc. Natl. Acad. Sci. U.S.A.* 115 1292–1297. 10.1073/pnas.1718316115 29358376 PMC5819444

[B19] Mogensen J. Klausen I. C. Pedersen A. K. Egeblad H. Bross P. Kruse T. A. (1999). Alpha-cardiac actin is a novel disease gene in familial hypertrophic cardiomyopathy. *J. Clin. Invest.* 103 R39–R43. 10.1172/JCI6460 10330430 PMC408458

[B20] Mogensen J. Perrot A. Andersen P. S. Havndrup O. Klausen I. C. Christiansen M. (2004). Clinical and genetic characteristics of α cardiac actin gene mutations in hypertrophic cardiomyopathy. *J. Med. Genet.* 41:e10. 10.1136/jmg.2004.018887 14729850 PMC1757257

[B21] Monserrat L. Hermida-Prieto M. Fernandez X. Rodríguez I. Dumont C. Cazón L. (2007). Mutation in the alpha-cardiac actin gene associated with apical hypertrophic cardiomyopathy, left ventricular non-compaction, and septal defects. *Eur. Heart J.* 28 1953–1961. 10.1093/eurheartj/ehm239 17611253

[B22] Morita H. Rehm H. L. Menesses A. McDonough B. Roberts A. E. Kucherlapati R. (2008). Shared genetic causes of cardiac hypertrophy in children and adults. *N. Engl. J. Med.* 358 1899–1908. 10.1056/NEJMoa075463 18403758 PMC2752150

[B23] Müller M. Mazur A. J. Behrmann E. Diensthuber R. P. Radke M. B. Qu Z. (2012). Functional characterization of the human α-cardiac actin mutations Y166C and M305L involved in hypertrophic cardiomyopathy. *Cell. Mol. Life Sci.* 69 3457–3479. 10.1007/s00018-012-1030-5 22643837 PMC11115188

[B24] Mundia M. M. Demers R. W. Chow M. L. Perieteanu A. A. Dawson J. F. (2012). Subdomain location of mutations in cardiac actin correlate with type of functional change. *PLoS One* 7:e36821. 10.1371/journal.pone.0036821 22590617 PMC3348139

[B25] Olivotto I. Girolami F. Ackerman M. J. Nistri S. Bos J. M. Zachara E. (2008). Myofilament protein gene mutation screening and outcome of patients with hypertrophic cardiomyopathy. *Mayo Clin. Proc.* 83 630–638. 10.4065/83.6.630 18533079

[B26] Olson T. M. (1998). Actin mutations in dilated cardiomyopathy, a heritable form of heart failure. *Science* 280 750–752. 10.1126/science.280.5364.750 9563954

[B27] Olson T. M. Doan T. P. Kishimoto N. Y. Whitby F. G. Ackerman M. J. Fananapazir L. (2000). Inherited and de novo mutations in the cardiac actin gene cause hypertrophic cardiomyopathy. *J. Mol. Cell. Cardiol.* 32 1687–1694. 10.1006/jmcc.2000.1204 10966831

[B28] Risi C. Eisner J. Belknap B. Heeley D. H. White H. D. Schröder G. F. (2017). Ca2+-induced movement of tropomyosin on native cardiac thin filaments revealed by cryoelectron microscopy. *Proc. Natl. Acad. Sci. U.S.A.* 114 6782–6787. 10.1073/pnas.1700868114 28607071 PMC5495243

[B29] Seidman J. G. Seidman C. (2001). The genetic basis for cardiomyopathy: from mutation identification to mechanistic paradigms. *Cell* 104 557–567. 10.1016/S0092-8674(01)00242-2 11239412

[B30] Semsarian C. Ingles J. Maron M. S. Maron B. J. (2015). New perspectives on the prevalence of hypertrophic cardiomyopathy. *J. Am. Coll. Cardiol.* 65 1249–1254. 10.1016/j.jacc.2015.01.019 25814232

[B31] Smith E. R. (2009). The Canadian heart health strategy and action plan. *Can. J. Cardiol.* 25 451–452. 19668778 10.1016/s0828-282x(09)70116-3 PMC2732371

[B32] Song W. Dyer E. Stuckey D. J. Copeland O. Leung M. C. Bayliss C. (2011). Molecular mechanism of the E99K mutation in cardiac actin (ACTC Gene) that causes apical hypertrophy in man and mouse. *J. Biol. Chem.* 286 27582–27593. 10.1074/jbc.M111.252320 21622575 PMC3149350

[B33] Song W. Vikhorev P. G. Kashyap M. N. Rowlands C. Ferenczi M. A. Woledge R. C. (2013). Mechanical and energetic properties of papillary muscle from ACTC E99K transgenic mouse models of hypertrophic cardiomyopathy. *Am. J. Physiol. Hear. Circ. Physiol.* 304 H1513–H1524. 10.1152/ajpheart.00951.2012 23604709 PMC3680724

[B34] Toko H. Takahashi H. Kayama Y. Oka T. Minamino T. Okada S. (2010). Ca2+/calmodulin-dependent kinase II causes heart failure by accumulation of p53 in dilated cardiomyopathy. *Circulation* 122 891–899. 10.1161/CIRCULATIONAHA.109.935296 20713897 PMC3226824

[B35] Van Driest S. L. Ellsworth E. G. Ommen S. R. Tajik A. J. Gersh B. J. Ackerman M. J. (2003). Prevalence and spectrum of thin filament mutations in an outpatient referral population with hypertrophic cardiomyopathy. *Circulation* 108 445–451. 10.1161/01.CIR.0000080896.52003.DF 12860912

[B36] Vang S. Corydon T. J. Børglum A. D. Scott M. D. Frydman J. Mogensen J. (2005). Actin mutations in hypertrophic and dilated cardiomyopathy cause inefficient protein folding and perturbed filament formation. *FEBS J.* 272 2037–2049. 10.1111/j.1742-4658.2005.04630.x 15819894

[B37] Viswanathan M. C. Schmidt W. Rynkiewicz M. J. Agarwal K. Gao J. Katz J. (2017). Distortion of the actin A-triad results in contractile disinhibition and cardiomyopathy. *Cell Rep.* 20 2612–2625. 10.1016/j.celrep.2017.08.070 28903042 PMC5902318

[B38] von der Ecken J. Heissler S. M. Pathan-Chhatbar S. Manstein D. J. Raunser S. (2016). Cryo-EM structure of a human cytoplasmic actomyosin complex at near-atomic resolution. *Nature* 534 724–728. 10.1038/nature18295 27324845

[B39] von der Ecken J. Müller M. Lehman W. Manstein D. J. Penczek P. A. Raunser S. (2015). Structure of the F-actin-tropomyosin complex. *Nature* 519 114–117. 10.1038/nature14033 25470062 PMC4477711

[B40] Walsh R. Thomson K. L. Ware J. S. Funke B. H. Woodley J. McGuire K. J. (2017). Reassessment of Mendelian gene pathogenicity using 7,855 cardiomyopathy cases and 60,706 reference samples. *Genet. Med.* 19 192–203. 10.1038/gim.2016.90 27532257 PMC5116235

[B41] Wong W. W. Doyle T. C. Cheung P. Olson T. M. Reisler E. (2001). Functional studies of yeast actin mutants corresponding to human cardiomyopathy mutations. *J. Muscle Res. Cell Motil.* 22 665–674. 10.1023/A:1016354308436 12222827

